# Survival Significance of Patients With Low Prostate-Specific Antigen and High-Grade Prostate Cancer After Radical Prostatectomy, External Beam Radiotherapy, or External Beam Radiotherapy With Brachytherapy

**DOI:** 10.3389/fonc.2019.00638

**Published:** 2019-07-19

**Authors:** Yadong Guo, Shiyu Mao, Aihong Zhang, Junfeng Zhang, Longsheng Wang, Ruiliang Wang, Wentao Zhang, Ziwei Zhang, Yuan Wu, Xuan Cao, Bin Yang, Xudong Yao

**Affiliations:** ^1^Department of Urology, Shanghai Tenth People's Hospital, Tongji University, Shanghai, China; ^2^Department of Medical Statistics, Tongji University School of Medicine, Shanghai, China

**Keywords:** prostate cancer, prostate specific antigen, gleason score, radical prostatectomy, radiotherapy, SEER data

## Abstract

**Objective:** This study compared survival of prostate cancer patients with low prostate specific antigen level (PSA ≤ 10 ng/ml) and high-grades of Gleason score (GS) of 8–10 with different treatment options (i.e., radical prostatectomy [RP], external beam radiotherapy [EBRT], or external beam radiotherapy with brachytherapy [EBRT+BT]).

**Materials and Methods:** The Surveillance, Epidemiology and End Results (SEER) database data (2004–2013), and overall survival (OS) and prostate cancer-specific mortality (PCSM), were evaluated using the Cox proportional hazards regression model and Fine and Gray competing risk model.

**Results:** The SEER data contained 9,114 patients, 4,175 of whom received RP, 4,114 received EBRT, and 825 received EBRT+BT with a median follow-up duration of 47 months. RP patients had significantly better OS than patients with EBRT and EBRT+BT (adjusted HR [AHR]: 3.36, 95% CI: 2.43–4.64, *P* < 0.001; AHR: 2.15, 95% CI: 1.32–3.48, *P* = 0.002; respectively). There was no statistical difference in PCSM between RP and EBRT+BT (AHR: 1.31, 95% CI: 0.61–2.80, *P* = 0.485), while EBRT had worse OS (*P* < 0.05). The subgroup analysis revealed that there was no statistical difference in prognosis of patients with age of >70 years old, or PSA levels of ≤ 2.5 ng/ml between RP and EBRT+BT (*P* > 0.05).

**Conclusion:** RP patients with low PSA levels and high GS had better OS compared to either EBRT or EBRT+BT, while RP and EBRT+BT resulted in significantly lower PCSM, compared to EBRT. Moreover, EBRT+BT and RP were associated with similar survival of patients with age of > 70 years old, or PSA levels of ≤ 2.5 ng/ml.

## Introduction

In the USA, prostate cancer has an estimated of 164,690 new cases and 29,430 cancer-related deaths in 2018 (1). Clinically, most prostate cancer patients are diagnosed as early staged low or intermediate-risk of disease, and merely one-third of American men are diagnosed with a high-risk disease (2), which has different treatment options, such as radical prostatectomy (RP) and radiation therapy (RT) (3). RT includes external beam radiation therapy (EBRT) and EBRT plus brachytherapy (EBRT + BT), and previous randomized trials have revealed that EBRT + BT have an advantage in the biochemical disease-free survival of patients, when compared with EBRT (4). Furthermore, other retrospective studies have also revealed better survival of patients after EBRT + BT (5). Recently, studies have reported that RP could improve cancer-specific mortality in patients with high-risk prostate cancer (6). However, another retrospective study revealed that there was no statistically significant difference in survival between patients receiving RP and EBRT + BT with or without androgen deprivation therapy (ADT) in high-risk localized prostate cancer patients after adjusting for the prognostic factors of prostate cancer (7). In addition, increased PSA level is an indicator of the poor prognosis (8, 9) and high-grade diseases. However, patients with high-grade and low PSA level had poorer prognosis (10). Furthermore, low PSA level and high-risk of disease may represent a unique entity with potential dedifferentiation biology (11). To date, there is still no uniform treatment standard for this group of patients. The present study selected these patients from the Surveillance, Epidemiology, and End Results (SEER) database, and assessed their survival significance after treatment with RP and RT (EBRT or EBRT + BT).

## Methods

### Database and Patient Selections

**Figure 1:**
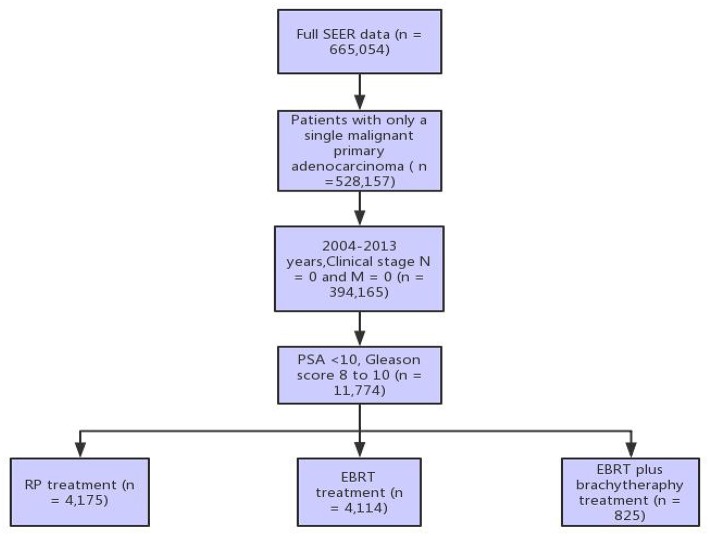
Illustration of the patient selection process.

The US SEER database, a population-based cancer registration system, provides different datasets on cancer incidence and survival by covering ~28% of US populations (https://seer.cancer.gov/). In the present study, the SEER^*^ Stat 8.3.5 software was utilized to query the data of patients diagnosed with primary prostate adenocarcinoma, had a pre-treatment PSA of ≤ 10 ng/dL, a GS of 8–10, and a clinical stage of N0 and M0 between 2004 and 2015. GS provided by the SEER program represents the highest GS found during a surgical or non-surgical biopsy. These patients received one of the three treatments (radical prostatectomy [RP], external beam radiotherapy [EBRT], or external beam radiotherapy with brachytherapy [EBRT+BT]), while patients who received prostate procedures and treatment before and after receiving RP were excluded. This dataset included 9,114 patients (Figure 1). The primary study endpoint was prostate cancer-specific mortality (PCSM) and overall survival (OS, death of any reason).

### Statistical Analysis

All statistical analyses were performed using Stata/MP 14.0 (StataCorp LP 4905 Lakeway Drive College Station, TX, USA) and R Studio v1.1.447 with survival and twang packages at a two-tailed level of significance of 0.05. The differences in categorical variables between groups were analyzed by chi-squared test, while normally distributed continuous variables were analyzed by one-way analysis of variance (ANOVA) test and the Kruskal-Wallis test for skewed continuous variables. The *P*-value of multiple comparisons was corrected using the Bonferroni method, while the propensity score was estimated by using the generalized boosted model (GBM), which analyzed the involvement of an iterative process with multiple regression trees to capture complex and non-linear relationships between the treatment assignment and pretreatment covariates without over-fitting the data, according to previous studies (12, 13). Moreover, the outcome of this model was a categorical variable, with 1 for RP, 2 for EBRT, and 3 for EBRT+BT. The co-variables of the model included race, marital status, age at diagnosis, years of diagnosis, PSA level, clinical T stage, and GS. Then, the mean and maximum standardized bias stopping rules were used to select the iteration that yielded the optimal balance to fit each GBM. The mnps () function in the twang package automated the propensity score and weight estimation process by running the GBM fitting algorithm for many iterations, and selecting the iteration to minimize the user-specified stopping rule. This produced weights from the selected model, and all the steps for all treatment groups were repeated. Moreover, for the standardized bias (absolute standardized mean difference) of each covariate, <0.20 was considered small, 0.40 was considered moderate, and 0.60 was considered large, according to a previous study (14). The estimated treatment effect on survival was analyzed using the Cox proportional regression model, according to previous studies.

In addition, Kaplan-Meier survival analysis was used to evaluate overall survival at 5 year and 10 year of follow-up and log-rank test generated *P*-values. Multivariate Cox regression was used to estimate the hazard ratios of overall survival between treatment groups with or without inverse propensity score of treatment weights, including the patient marital status, age at diagnosis, year of diagnosis, race, PSA, clinical T stage, and Gleason score in the Cox regression model along with the treatment indicator (therapy). Similarly competing risks regression was used to estimate the hazard ratios of prostate cancer-specific mortality between treatment groups with or without inverse propensity score of treatment weights, including patient marital status, age at diagnosis, year of diagnosis, race, PSA, clinical T stage, and Gleason score in the Fine-Gray model at the same time.

## Results

### Patients Characteristics

The SEER database had 9,114 prostate cancer patients with a GS of 8–10 and a pre-treatment PSA level of ≤ 10 ng/dL, among which 4,175 (45.8%) received RP, 4,114 (45.1%) received EBRT, and 825 (9.1%) received EBRT + BT with a median follow-up duration of 47 months (interquartile range [IQR], 34–60), 47 months (IQR, 34–60) for RP, 47 months (IQR, 33–60) for EBRT, and 51 months (IQR, 37–62) for EBRT + BT. Furthermore, the median age of patients was 67 years old (IQR, 62–73), 64 years old (IQR, 59–68) for RP, 71 years old (IQR, 66–76) for EBRT, and 68 years old (IQR, 62–73) for EBRT+BT (Table 1; Supplementary Table 1 and Supplementary Figure 1).

**Table 1:** Clinicopathological features of prostate cancer patients with low PSA levels and high Gleason scores.

**Clinical characteristics**	**Unweighted**, ***n*** **(%)**			***P*****-value**		
	**RP (*n* = 4,175)**	**EBRT (*n* = 4,114)**	**EBRT+BT (*n* = 825)**	**EBRT vs. RP**	**EBRT+BT vs. RP**	**EBRT+BT vs. EBRT**
Age at diagnosis				<0.001	<0.001	<0.001
Mean (median)	63.7 (64.0)	70.6 (71.0)	67.3 (68.0)			
[range], year	[59.0–68.0]	[66.0–76.0]	[62.0–73.0]			
PSA level				<0.001	<0.001	>0.999
Mean (median)	5.9 (5.7)	6.3 (6.3)	6.3 (6.1)			
[range], ng/mL	[4.6–7.2]	[4.8–7.9]	[4.9–7.8]			
Marital status				<0.001	<0.001	0.228
Married	3,176 (76.1)	2,747 (66.8)	577 (70.0)			
Divorced/widowed	405 (9.8)	623 (15.1)	99 (12.0)			
Singled	376 (9.0)	333 (8.1)	74 (9.0)			
Unknown	218 (5.2)	411 (10.0)	75 (9.1)			
Race				<0.001	<0.001	0.001
White	3,298 (79.0)	3,189 (77.5)	595 (72.1)			
Black	524 (12.6)	600 (14.6)	161 (19.5)			
Other	309 (7.4)	233 (5.7)	58 (7.0)			
Unknown	44 (1.1)	92 (2.2)	11 (1.3)			
AJCC T stage				<0.001	<0.001	0.012
T1	19 (0.5)	2,241 (54.5)	500 (60.6)			
T2	2,608 (62.5)	1,612 (39.2)	278 (33.7)			
T3	1,448 (34.7)	228 (5.5)	45 (5.5)			
T4	100 (2.4)	33 (0.8)	2 (0.2)			
Gleason score				<0.001	>0.99	0.003
8	2,998 (71.8)	2,601 (63.2)	573 (69.5)			
9	1,116 (26.7)	1,380 (33.5)	238 (28.8)			
10	61 (1.5)	133 (3.2)	14 (1.7)			

*RP, radical prostatectomy; EBRT, external beam radiotherapy; EBRT+BT, external beam radiotherapy with brachytherapy boost; PSA, prostate-specific antigen*.

### Association of Treatment Options With OS and PCSM of Patients

**Figure 2:**
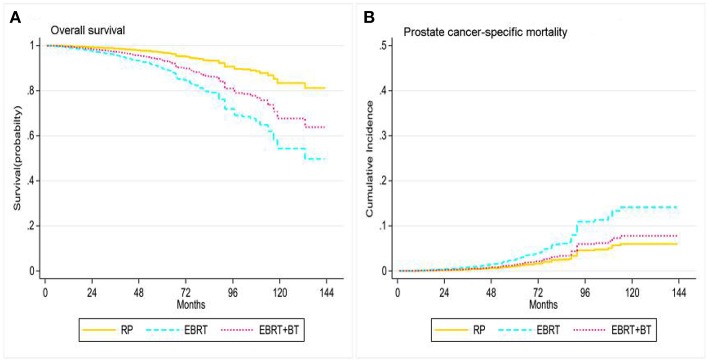
Adjusted survival curves for overall survival **(A)** and prostate cancer-specific mortality **(B)** by RP, EBRT, and EBRT+BT treatment options after weighting (adjusted curves after stratified by RP, EBRT, and EBRT+BT treatment options were generated by adding marital status, race, age at diagnosis, disease stage, PSA level, and GS into the Cox proportional hazards model or competing risks regression model, respectively).

Treatment options were associated with OS and PCSM of patients and the 3-, 5-, and 10-year OS of patients were as follows: 98.4, 96.8, and 67.5% for RP, respectively; 95.1, 87.3, and 58.0% for EBRT, respectively; 96.7, 92.8, and 61.5% for EBRT+BT, respectively. Furthermore, the 3-, 5-, and 10-year PCSM of patients were as follows: 0.5, 1.4, and 16.3% for RP, respectively; 1.4, 4.8, and 23.7% for EBRT, respectively; 0.8, 2.3, and 6.5% for EBRT+BT, respectively (Figure 2 and Table 2). The multivariate Cox regression analysis after adjusting for the patient's marital status, age at diagnosis, race, PSA level, clinical T stage, and GS revealed that RP was associated with better OS, compared to EBRT or EBRT+BT (adjusted HR [AHR]: 3.36, 95% CI: 2.43–4.64, *P* < 0.001; AHR: 2.15, 95% CI: 1.32–3.48, *P* = 0.002; respectively; Table 3). However, in the competitive risk model after adjusting for the patient's marital status, age at diagnosis, race, PSA level, clinical T stage, and GS, no significant difference was found in PCSM for patients treated with RP vs. EBRT + BT (AHR: 1.31, 95% CI: 0.61–2.80, *P* = 0.485). Moreover, RP was associated with significantly better PCSM, compared to EBRT (AHR: 2.46, 95% CI: 1.45–4.18, *P* = 0.001; Table 3).

**Table 2:** The 3-, 5-, and 10-year overall survival and prostate cancer-specific mortality of patients after RP, EBRT, and EBRT+BT.

**Therapy**	***n* (%)**	**Unweighted (%)**			**Weighted (%)**		
		**3-year (95% CI)**	**5-year (95% CI)**	**10-year (95% CI)**	**3-year (95% CI)**	**5-year (95% CI)**	**10-year (95% CI)**
**OVERALL SURVIVAL USING KAPLAN MEIER ANALYSIS**							
RP	4,175 (45.8)	98.3 (97.9–98.7)	96.2 (95.3–96.9)	73.5 (54.8–85.5)	98.4 (98.1–98.7)	96.8 (96.2–97.2)	67.5 (49.3–80.4)
EBRT	4,114 (45.1)	94.0 (93.2–94.6)	86.3 (86.2–87.6)	54.7 (39.0–68.0)	95.1 (94.6–95.6)	87.3 (86.3–88.2)	58.0 (49.4–65.6)
EBRT+BT	825 (9.1)	96.8 (95.3–97.9)	92.5 (0.89.8–94.5)	66.5 (35.5–85.2)	96.7 (96.3–97.1)	92.8 (92.1–93.5)	61.5 (47.3–72.9)
**PROSTATE CANCER-SPECIFIC MORTALITY**							
RP	4,175 (45.8)	6 (4–9)	16 (12–22)	17.1 (8–34.3)	5 (4–7)	1.4 (1.1–1.7)	16.3 (8.4–30.1)
EBRT	4,114 (45.1)	1.9 (1.5–2.4)	5.3 (4.4–6.4)	20.8 (13–32.4)	1.4 (1.2–1.7)	4.8 (4.2–5.5)	23.7 (18.6–29.9)
EBRT+BT	825 (9.1)	1 (0.5–2)	2.6 (1.5–4.4)	8.4 (3.6–18.8)	0.8 (0.6–1)	2.3 (1.9–2.8)	6.5 (4.8–8.7)

**Table 3:** Proportional hazards regression model for the association of different treatments with overall survival and prostate cancer-specific mortality.

**Covariate^**a**^**	**Cox proportional hazards regression overall survival**				**Competing risk regression prostate cancer-specific mortality**			
	**Unweighted**		**Weighted**		**Unweighted**		**Weighted**	
	**Survival, HR (95% CI)**	***P*-value**	**Survival, HR (95% CI)**	***P*-value**	**Survival, SHR (95% CI)**	***P*-value**	**Survival, SHR (95% CI)**	***P*-value**
RP	1 (Reference)		1 (Reference)		1 (Reference)		1 (Reference)	
EBRT	3.29 (2.56–4.19)	<0.001	3.36 (2.43–4.64)	<0.001	2.77 (1.91–3.40)	<0.001	2.46 (1.45–4.18)	0.001
EBRT+BT	2.03 (1.44–2.88)	<0.001	2.15 (1.32–3.48)	0.002	1.76 (0.98–3.14)	0.057	1.31 (0.61–2.80)	0.485

a *The multivariate Cox regression and competing risk regression derived-hazard ratios are adjusted for age at diagnosis, marital status, race, Gleason score, disease stage, and PSA level*.

### Association of Treatment Options With OS and PCSM of Patients Stratified by Age and PSA Level

**Figure 3:**
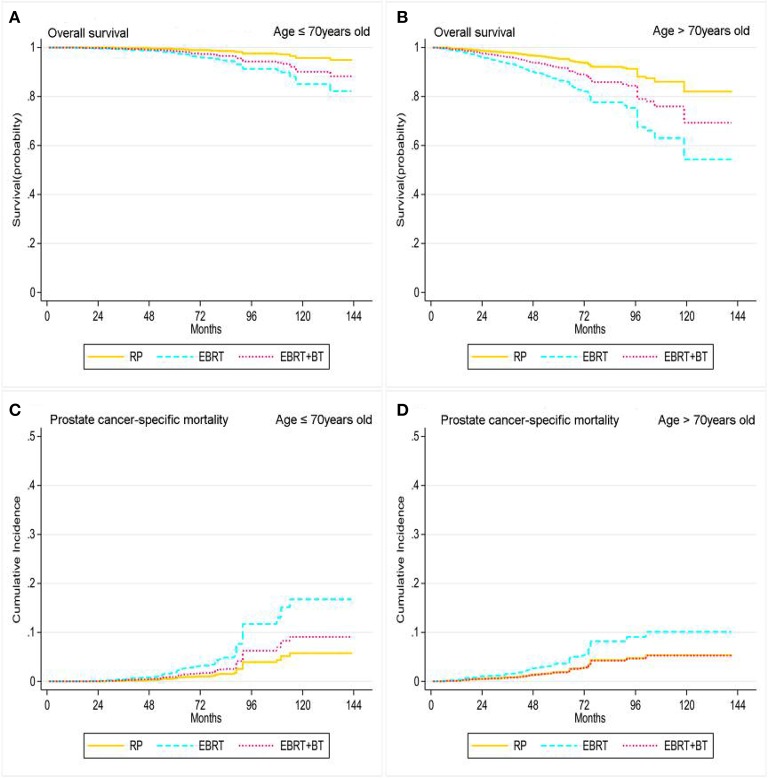
Adjusted survival curves for overall survival (**A**, age ≤ 70 years old; **B**, age > 70 years old) and prostate cancer-specific mortality (**C**, age ≤ 70 years old; **D**, age > 70 years old) by RP, EBRT, and EBRT+BT treatment options after weighting in age subgroups after weighting (adjusted curves after stratified by RP, EBRT, and EBRT+BT treatment options were generated by adding marital status, race, at diagnosis, disease stage, PSA level, and GS into the Cox proportional hazards model or competing risks regression model, respectively).

Treatment options were associated with the OS and PCSM of patients stratified by age and PSA level. The Cox proportional hazards regression and competing risk model after adjusting for the patient's marital status, race, PSA level, clinical T stage, and GS found patients who were ≤70 years old after RP had significantly better OS compared to patients who received EBRT and EBRT+BT (*P* < 0.05). However, there was no statistical difference in PCSM between RP and EBRT+BT (AHR: 1.63, 95% CI: 0.69–3.86; *P* = 0.266), and there was no statistical difference in OS for patients who were >70 years old between RP and EBRT+BT (AHR: 1.84, 95% CI: 0.95–3.57, *P* = 0.071), although patients who were >70 years old and received RP had a significant increase in OS compared with EBRT (*P* < 0.001; Table 4 and Figure 3). Moreover, there was no statistical difference in PCSM occurring among all three-treatment groups (*P* > 0.05; Table 4 and Figure 3).

**Table 4:** Proportional hazards regression model for the association of different treatments with overall survival and prostate cancer-specific mortality stratified by Gleason score, age, and PSA level.

**Covariate**	**Cox proportional hazards regression overall survival**				**Competing risk regression prostate cancer-specific mortality**			
	**Unweighted**		**Weighted**		**Unweighted**		**Weighted**	
	**Survival, HR (95% CI)**	***P*-value**	**Survival, HR (95% CI)**	***P*-value**	**Survival, SHR (95% CI)**	***P*-value**	**Survival, SHR (95% CI)**	***P*-value**
^a^Age ≤ 70 years old								
RP	1 (Reference)		1 (Reference)		1 (Reference)		1 (Reference)	
EBRT	3.10 (2.21–4.34)	<0.001	3.65 (2.58–5.16)	<0.001	3.15 (1.87–5.31)	<0.001	3.12 (1.87–5.18)	<0.001
EBRT+BT	1.90 (1.15–3.14)	0.012	2.35 (1.24–4.45)	0.009	1.65 (0.70–3.90)	0.25	1.63 (0.69–3.86)	0.266
^a^Age > 70 years old								
RP	1 (Reference)		1 (Reference)		1 (Reference)		1 (Reference)	
EBRT	3.17 (2.17–4.63)	<0.001	3.07 (1.82–5.17)	<0.001	2.29 (1.31–3.99)	0.004	1.94 (0.87–4.32)	0.105
EBRT+BT	1.86 (1.10–3.13)	0.02	1.84 (0.95–3.57)	0.071	1.04 (0.42–2.59)	0.934	0.98 (0.30–3.15)	0.97
^b^PSA ≤ 2.5 ng/ml								
RP	1 (Reference)		1 (Reference)		1 (Reference)		1 (Reference)	
EBRT	2.28 (0.84–6.21)	0.106	4.00 (1.44–11.13)	0.008	2.45 (0.71–8.42)	0.154	5.13 (1.34–19.65)	0.017
EBRT+BT	0.71 (0.76–6.68)	0.765	0.58 (0.09–3.61)	0.556	1.01 (0.18–5.53)	0.993	1.27 (0.25–6.59)	0.774
^b^PSA 2.5–4 ng/ml								
RP	1 (Reference)		1 (Reference)		1 (Reference)		1 (Reference)	
EBRT	2.91 (1.46–5.80)	0.002	2.89 (1.50–5.55)	0.001	4.94 (1.76–13.86)	0.002	9.94 (1.51–65.50)	0.017
EBRT+BT	2.49 (0.89–6.96)	0.081	4.33 (1.26–14.8)	0.02	4.56 (0.92–22.58)	0.063	7.29 (0.58–92.03)	0.125
^b^PSA > 4 ng/ml								
RP	1 (Reference)		1 (Reference)		1 (Reference)		1 (Reference)	
EBRT	3.70 (2.52–5.43)	<0.001	3.48 (2.40–5.06)	<0.001	2.65 (1.74–4.04)	<0.001	2.19 (1.21–3.97)	0.01
EBRT+BT	2.00 (1.21–3.30)	0.007	2.02 (1.22–3.35)	0.007	1.63 (0.84–3.15)	0.14	1.22 (0.51–2.89)	0.657

a *The multivariate Cox regression and competing risk regression derived-hazard ratios are adjusted for marital status, race, Gleason score, disease stage, and PSA level*.

b *The multivariate Cox regression and competing risk regression derived-hazard ratios are adjusted for age at diagnosis, marital status, race, Gleason score, and disease stage*.

**Figure 4:**
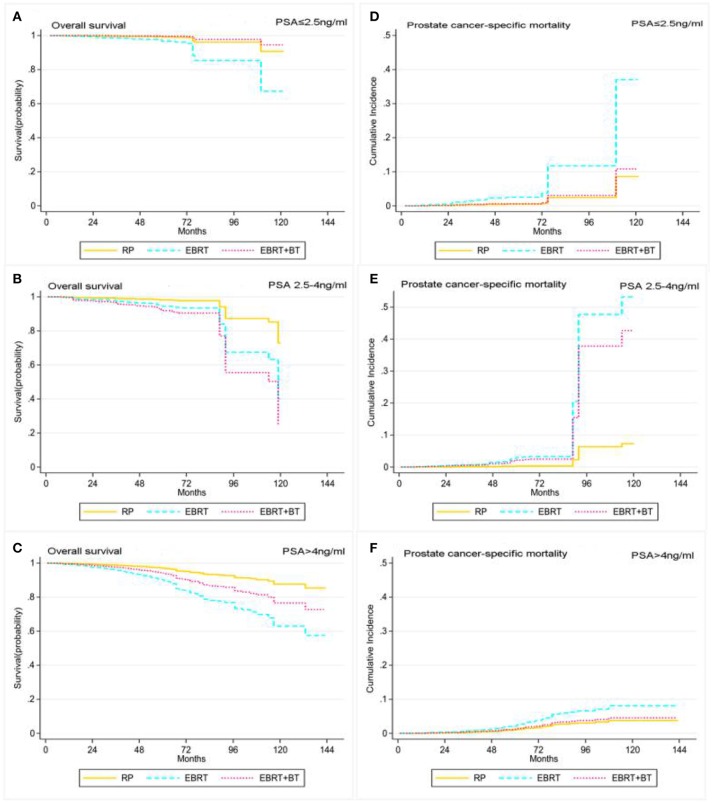
Adjusted survival curves for overall survival (**A**, PSA ≤ 2.5 ng/ml; **B**, PSA 2.5–4 ng/ml; **C**, PSA > 4 ng/ml) and prostate cancer-specific mortality (**D**, PSA ≤ 2.5 ng/ml; **E**, PSA 2.5–4 ng/ml; **F**, PSA > 4 ng/ml) by RP, EBRT, and EBRT+BT treatment options after weighting in PSA level subgroups after weighting (adjusted curves after stratified by RP, EBRT, and EBRT+BT treatment options were generated by adding marital status, race, age at diagnosis, disease stage, and GS into the Cox proportional hazards model or competing risks regression model, respectively).

In addition, the Cox proportional hazards regression and competing risk model, after adjusting for the patient's marital status, age at diagnosis, race, clinical T stage, and GS, found RP and EBRT+BT did not yield any statistical differences in OS and PCSM for patients with PSA levels of ≤2.5 ng/ml (*P* > 0.05), but EBRT contributed to worsen the OS and PCSM of patients with a PSA level of ≤2.5 ng/ml compared to patients who received RP (*P* < 0.05; Table 4 and Figure 4). Furthermore, patients with PSA levels of 2.5–4 ng/ml after RP had significantly better OS compared to patients who received EBRT and EBRT+BT (AHR: 2.89, 95% CI: 1.50–5.55, *P* = 0.001; AHR: 4.33) 95% CI: 1.26–14.8, *P* = 0.02; Table 4 and Figure 4). Moreover, there was no statistical significance in PCSM for patients with PSA levels of 2.5–4 ng/ml after RP and EBRT + BT (*P* > 0.05), and patients with PSA levels of 2.5–4 ng/ml after EBRT had worse PCSM compared to patients who received RP (*P* < 0.05; Table 4 and Figure 4). Sensitivity analyses showed that prognosis of these three treatments of PSA levels of the 2.5–4 ng/ml group was similar to that of the PSA levels of >4 ng/ml group (Table 4 and Figure 4).

## Discussion

Recently, increasing attention has focused on treatment of high-risk localized prostate cancer, especially for the subgroup of high-risk localized prostate cancer (15, 16). Moreover, detection of PSA levels has been widely used to screen prostate cancer and monitor disease progression, although PSA levels may not always represent the degree of prostate cancer malignancy (17). Prostate cancer with low PSA level, but high disease grade, provides a unique and aggressive entity in clinic, and the risk of patient death has more than doubled, when compared to other high-risk diseases, according to the NCCN (11). Although the treatment of these specific high-risk patients with low PSA levels is important, there have been no reports in literature at present. Thus, in the present study, the survival significance of patients with low PSA level, but with high GS for prostate cancer after RP, EBRT, or EBRT plus BT, was assessed for future guidance on the treatment of these kind of patients in clinic. The present data revealed that patients who received RP had significantly better OS, when compared to patients who received EBRT or EBRT+BT. However, EBRT led to worse OS, although there was no statistical difference in PCSM between RP and EBRT+BT. The present subgroup analysis revealed that there was no statistical significance in OS and PCSM between RP and EBRT+BT in patients with age of >70 years old, or PSA level of ≤2.5 ng/ml. Furthermore, it could be concluded that RP of patients with low PSA level and high GS had better OS, when compared to patients who received either EBRT, or EBRT+BT, and that RP and EBRT+BT led to significantly lower PCSM, when compared to EBRT, suggesting that EBRT+BT might be an alternative option for treating patients with age of >70 years old, or PSA of ≤2.5 ng/ml.

The present data assessed a large cohort of patient samples, and the statistical power was strong, which could minimize significant baseline differences in clinical and demographic variables among these three different treatment options (RP, EBRT, and EBRT+BT) for association with the prognosis. A previous meta-analysis conducted by Wallis et al. revealed that surgery could have reduced the overall and prostate cancer-specific mortality of patients with locally high-risk prostate cancer (18), while Ennis et al. revealed that there was no survival significance in patients with high-risk localized prostate cancer after treatment with RP or EBRT+BT with or without ADT (7). In the present study, the investigators were able to verify the effectiveness of RP in treating high-risk prostate cancer patients. Other studies have reported that EBRT+BT was better in controlling biochemical recurrence and survival, when compared with EBRT (4, 5), and it was further confirmed by the present data that EBRT+BT was associated with longer 10-year cancer specific survival, when compared to RP and EBRT. Prostate cancer-specific mortality is more frequent than other causes, which may explain the improvement in survival of patients after EBRT + BT. Indeed, randomized trials and retrospective studies have reported similar prostate cancer-specific mortality in EBRT + BT and RP (19, 20). The subgroup analysis of this cohort of patients was also conducted. Since patients in the radiotherapy cohort are usually older and have more comorbidities, a subgroup analysis stratified by the age of patients was thereby performed, while a patient age of 70 years old, as one of the optimal cut-points, was detected using the Optimal Binning procedure that discretizes variable age with respect to the guide variable GS that “supervises” the binning process. In addition, it was found that RP still had a better OS in patients who were ≤ 70 years old, when compared with radiotherapy, while EBRT+BT and RP had the same prognosis in patients with >70 years old. A previous study performed by Huang et al. compared the effects of surgery and radiation therapy on the cancer-specific mortality of locally high-grade prostate cancer patients who were <60 years old, and revealed a significant difference in survival between initial surgery and radiation therapy (16). In addition, patients with high-grade (GS 8–10) localized prostate cancer, a PSA of ≤2.5 and 2.5–4 ng/mL was more likely to have cancer-specific death, when compared to PSA levels between 4 and 10 ng/ml (10). In the present study, patients were stratified for PSA levels of 2.5 and 4 as a cutoff value, and it was found that RP and EBRT+BT treatments contributed to the better prognosis of patients with a PSA of ≤2.5 ng/mL. However, treated patients with PSA levels of 2.5–4 and 4–10 ng/ml, who had undergone RP, had significantly increased OS, when compared to those who received EBRT and EBRT+BT. Although, patients after RP and EBRT+BT had no significant difference in PCSM. Furthermore, patients with a high-grade, but low-PSA prostate cancer usually have poor prognosis and poorly differentiated tumors, thereby leading to low sensitivity to traditional ADT (11), and making RP a better choice of treatment.

The primary clinical significance of the present data was the discovery showing that RP was the treatment option for patients with high-grade, but low-PSA, prostate cancer, while EBRT+BT is an alternative option for the treatment of patients with an age of >70 years old, or a PSA level of ≤2.5 ng/ml. However, in the present study, cases of subsequent treatment with RP were excluded. It is possible that RP shows advantages in treating these kind of patients: (1) simple surgically resected tissue specimens are better for assessing the extent of cancer progression, and the follow-up data will guide further treatments, which is similar to RT in improving the survival of patients (21); (2) surgery could also reduce tumor burden for better local control of the disease and improving systemic treatment response (22); (3) The surgical resection of tissue lesions reduces PSA levels more rapidly, thereby improving physiological conditions for better disease-free survival, when compared with RT; (4) surgery leads to less cytotoxic side effects and comorbidities (23, 24).

However, the present study does have some limitations. For example, it is a retrospective study, and even after adjusting for propensity scores, bias may still exist, when compared to treatment modalities and patient baseline characteristics. Furthermore, the SEER database does not provide data on treatment details, such as ADT, duration, radiation dosage, duration, and comorbidities. In addition, the present study lacked a toxicity data for analysis, which is also a shortfall, because RP and EBRT have different toxicity characteristics (25). Therefore, future prospective studies are needed to determine the long-term outcome of these treatments.

## Conclusion

The present study demonstrated that the treatment of patients with low PSA, but with high-grade prostate cancer, with radical prostatectomy, contributed to the significant increase in OS, when compared with EBRT and EBRT+BT. Whereas, radical prostatectomy and EBRT+BT were associated with significantly lower PCSM, when compared to EBRT. EBRT+BT could be an alternative option in the treatment of patients with an age >70 years old, or PSA levels of ≤2.5 ng/ml.

## Data Availability

The raw data supporting the conclusions of this manuscript will be made available by the authors, without undue reservation, to any qualified researcher.

## Author Contributions

YG and XC: data curation. SM and JZ: formal analysis. BY and XY: funding acquisition and supervision. LW and RW: investigation. AZ and WZ: methodology. XY: project administration and resources. AZ and ZZ: software. YW: validation. BY: visualization. YG: writing—original draft and review and editing.

### Conflict of Interest Statement

The authors declare that the research was conducted in the absence of any commercial or financial relationships that could be construed as a potential conflict of interest.
