# Supplementary material for: Survival Significance of Patients With Low Prostate-Specific Antigen and High-Grade Prostate Cancer After Radical Prostatectomy, External Beam Radiotherapy, or External Beam Radiotherapy With Brachytherapy
Source: Front Oncol. 2019 Jul 19;9:638. doi: 10.3389/fonc.2019.00638 (PMC6659440; doi:10.3389/fonc.2019.00638)
Supplement: Supplementary file 1 [file Data_Sheet_1.doc]

Supplementary Table 1. Clinicopathological characteristics of patients with low-PSA and high-grade prostate cancer

| Pretreatment covariate | Unweighted means | | | Unweighted standard bias | | | Weighted means | | | Weighted standard bias | | |
| --- | --- | --- | --- | --- | --- | --- | --- | --- | --- | --- | --- | --- |
|  | RP | EBRT | EBRT+BT | RP VS EBRT | EBRT VS EBRT+BT | EBRT+BT VS RP | RP | EBRT | EBRT+BT | RP VS EBRT | EBRT VS EBRT+BT | EBRT+BT VS RP |
| Marital status |  |  |  |  |  |  |  |  |  |  |  |  |
| Married | 0.761 | 0.668 | 0.699 | 0.215 | 0.073 | 0.142 | 0.661 | 0.706 | 0.696 | 0.103 | 0.023 | 0.08 |
| Divorced/widowed | 0.097 | 0.151 | 0.12 | 0.181 | 0.104 | 0.076 | 0.102 | 0.129 | 0.122 | 0.091 | 0.024 | 0.066 |
| singled | 0.09 | 0.081 | 0.09 | 0.032 | 0.031 | 0.001 | 0.085 | 0.087 | 0.102 | 0.005 | 0.052 | 0.057 |
| Unknown | 0.052 | 0.1 | 0.091 | 0.203 | 0.038 | 0.165 | 0.152 | 0.079 | 0.081 | 0.313 | 0.01 | 0.302 |
| Age at diagnosis | 63.706 | 70.633 | 67.333 | 0.885 | 0.422 | 0.463 | 65.819 | 67.639 | 67.073 | 0.232 | 0.072 | 0.16 |
| Race |  |  |  |  |  |  |  |  |  |  |  |  |
| White | 0.79 | 0.775 | 0.721 | 0.036 | 0.13 | 0.166 | 0.794 | 0.783 | 0.789 | 0.028 | 0.016 | 0.012 |
| Black | 0.126 | 0.146 | 0.195 | 0.059 | 0.143 | 0.203 | 0.097 | 0.145 | 0.137 | 0.141 | 0.024 | 0.117 |
| Other | 0.074 | 0.057 | 0.07 | 0.067 | 0.052 | 0.014 | 0.052 | 0.056 | 0.061 | 0.016 | 0.02 | 0.036 |
| Unkown | 0.011 | 0.022 | 0.013 | 0.113 | 0.087 | 0.027 | 0.057 | 0.016 | 0.013 | 0.394 | 0.031 | 0.425 |
| AJCC T stage |  |  |  |  |  |  |  |  |  |  |  |  |
| T1 | 0.005 | 0.545 | 0.606 | 1.771 | 0.201 | 1.972 | 0.23 | 0.319 | 0.321 | 0.293 | 0.005 | 0.298 |
| T2 | 0.625 | 0.392 | 0.337 | 0.471 | 0.111 | 0.582 | 0.544 | 0.498 | 0.5 | 0.093 | 0.004 | 0.089 |
| T3 | 0.347 | 0.055 | 0.055 | 0.637 | 0.002 | 0.639 | 0.21 | 0.171 | 0.169 | 0.084 | 0.006 | 0.09 |
| T4 | 0.024 | 0.008 | 0.002 | 0.113 | 0.04 | 0.152 | 0.017 | 0.012 | 0.011 | 0.033 | 0.008 | 0.041 |
| PSA | 5.91 | 6.293 | 6.254 | 0.19 | 0.019 | 0.171 | 6.12 | 6.129 | 6.152 | 0.004 | 0.011 | 0.016 |
| Gleason score |  |  |  |  |  |  |  |  |  |  |  |  |
| 8 | 0.718 | 0.632 | 0.695 | 0.19 | 0.138 | 0.052 | 0.663 | 0.662 | 0.668 | 0.003 | 0.013 | 0.009 |
| 9 | 0.267 | 0.335 | 0.288 | 0.153 | 0.106 | 0.048 | 0.323 | 0.314 | 0.315 | 0.02 | 0.002 | 0.017 |
| 10 | 0.015 | 0.032 | 0.017 | 0.146 | 0.126 | 0.019 | 0.014 | 0.024 | 0.017 | 0.084 | 0.056 | 0.028 |

EBRT+BT, external beam radiotherapy with brachytherapy boost; PSA, prostate- specific antigen.

Supplementary Table 1 shows Clinicopathological characteristics of patients with low-PSA and high-grade prostate cancer for pre-weighting and weighted. For the pre-weighting, patients who underwent RP had higher proportion of Married status, T2 and T3 stage, lower proportion of unknown marital status, T1 stage and lower mean age at diagnosis than the RT group. Weighting reduces the standardized bias greatly, but the values of SBmax for unknown marital status, age at diagnosis, unknown race, and T1 stage between RP and RT group still remain above 0.20. So all pretreatment covariables were included in the model to estimate the confounding effect in addition to the variable therapy when weighted Cox proportional hazards regression were performed.


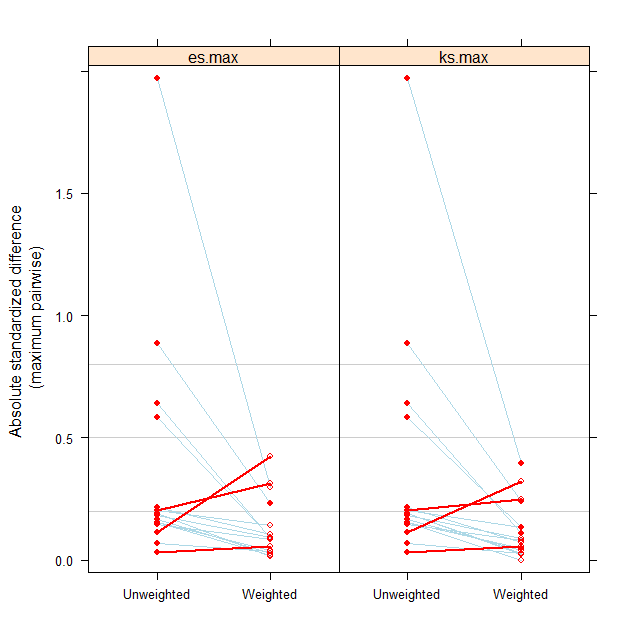


Supplementary Figure 1. Effective size plots to assess the balance between groups on pretreatment variables before and after ATE weighting. The dots on the left panel are the absolute standardized difference (maximum pairwise) or standardized bias (SBmax) for the unweighted samples. The dots on the right panel are the values for the weighted samples. The lines connect the values for the same pretreatment variable before and after weighting. A closed red square indicates a covariate for which the difference between treatment group means is statistically significant. An open red square indicates none of the differences is statistically significant. A red line identifies a variable for which the standardized bias or effect size increases with weighting; a blue line identifies a variable for which balance improves with weighting.
